# The Operative Surgery of Labyrinthitis

**Published:** 1908-04-04

**Authors:** 


### Progress in Otology.

### THE OPERATIVE SURGERY OF LABYRINTHITIS.

Brief Abstract of a Paper based upon an Experience of 30 Cases, by C. ERNEST WEST, F.R.C.S., Assistant Aural Surgeon, St. Bartholomew's Hospital, and SYDNEY SCOTT, M.S., F.R.C.S., Chief Assistant, Aural Department, St. Bartholomew's Hospital, and Aural Surgeon to the Evelina Hospital for Sick Children, read before the Otological Section of the Royal Society of Medicine, March 7, 1908.

After tracing briefly the history of the rise of labyrinthine surgery, the authors proceeded to give a detailed description of the surgical anatomy of the labyrinth. They next considered the morbid anatomy and course of infective disease of the labyrinth, exemplified by their cases, of which 26 were operated upon. In two out of the four cases, in which no operation on the labyrinth was performed, opportunities of investigating the labyrinth had arisen post-mortem.

After mentioning the infective organisms found, the paths of invasion of the labyrinth by middle ear disease were described, together with the changes in the labyrinth, and the spread of infection from the labyrinth to the brain and meninges.

The special danger of labyrinthitis was shown to lie in the extension of infection along the nerves in the internal auditory meatus, with the production of meningitis, more rarely cerebellar abscess, and in one case, acute internal hydrocephalus.

It was pointed out, that many cases of gross labyrinthine involvement, present a total absence of any characteristic symptoms, though, as shown by the record of cases, they by no means escape the dangers of the condition. Where symptoms declare themselves, vertigo of a definite type, most frequently horizontal, is almost always present. Important, though subsidiary, symptoms are vomiting, tinnitus, and deafness, while in acute cases local pain, headache, and general constitutional disturbance are marked features.

Symptoms, morbid anatomy, and operative results alike indicate the opening and adequate drainage of the vestibule as of the first importance. The tympanic aspect of this cavity is subdivided by the course of the facial nerve into upper and lower segments, through either of which the vestibule may be opened. In the superior opening entrance is made along the outer limb of the external semicircular canal and its ampulla, the ampullary end of the superior canal is opened, and the roof of the vestibule removed. This operation the authors have named Superior Vestibulotomy. They discarded the operation after performing it in two cases, one of which proved fatal, owing to the incomplete drainage which it afforded.

In the inferior opening, that portion of the outer wall of the vestibule below the facial nerve is removed. This operation is what the authors call Inferior Vestibulotomy. It may be frequently extended with advantage by the removal of the outer wall of the first half turn of the cochlea, a procedure which affords freer drainage. Where the superior opening of the vestibule is necessitated by the locality of the disease, it should be combined with Inferior Vestibulotomy, and this operation is named Double Vestibulotomy. Superior Vestibulotomy may be extended by complete removal of the semi-circular canals, Inferior Vestibulotomy by total removal of the cochlea. The term Extirpation of the Labyrinth is reserved for the complete operation in which both these procedures are combined. It is only rarely called for, by the most extensive disease.

In cases of local disease of the external semicircular canal without evidence of involvement of the ampulla or vestibule, local curettage sometimes proved to be adequate treatment. In an important group of cases sequestra of the labyrinth are discovered during operation on the middle ear. Such sequestra may be deeply buried in the petrous portion, and present great difficulties in their removal.

The following special tests are of value in eliciting evidence of labyrinthine disease: (1) Rombergism, (2) Gait, (3) Rotation phenomena. The indication for operation of the labyrinth is considered to be the evidence of infective labyrinthitis, resting either upon obvious symptoms and the response to special tests, or upon the discovery of gross labyrinthine disease, during the course of the radical mastoid operation. Having regard to the mortality of infective labyrinthitis, the results of operation are satisfactory. Two cases died of cerebellar abscess. In one case only could the fatal issue be ascribed to an operation on the labyrinth, which was incompletely drained.

Where vertigo or other labyrinthine symptom was present, complete relief was in all cases obtained by the operation, and there was no case of permanent facial paralysis following this procedure.

* Paper to be published in full, with illustrations, in the "Proceedings of the Royal Society of Medicine," April 1908.

